# Diverging trends in health at older ages in England, 2004–2024: evidence from the English Longitudinal Study of Ageing

**DOI:** 10.64898/2026.07.13.26357914

**Published:** 2026-07-16

**Authors:** Wu Jiawei, Glaser Karen, Price Debora, Di Gessa Giorgio

## Abstract

**Background:**

Given uncertainty about whether later-life health at similar ages is improving over time, we examined trends across multiple health domains.

**Methods:**

We analysed data from community-dwelling adults aged 50 and older in the English Longitudinal Study of Ageing in 2004/05, 2012/13, and 2023/24 (main survey: N=8389, 8549, and 6090, respectively). Outcomes included self-rated health, limiting long-standing illness, pain, mobility limitations, cardiometabolic and chronic conditions, obesity, inflammation, mental health, quality of life and memory. Weighted pooled modified Poisson and linear regressions compared outcomes over time, overall, and by age group and education, with additional adjustment for sex and wealth.

**Results:**

Adjusted estimates showed divergent trends. Fair/poor self-rated health increased from 27% to 34%, and any pain from 37% to 47%, whereas mobility impairments declined from 58% to 52%. Self-reported high cholesterol increased from 19% to 39%, while biomarker-defined high cholesterol declined from 78% to 54%; diabetes increased on both measures. Psychiatric problems increased from 6% to 10%, quality of life declined, and memory improved. However, trends differed by age and education, particularly for limiting long-standing illness, mobility limitations, cholesterol biomarkers, and mental health, indicating that aggregate trends masked unevenly distributed changes.

**Conclusion:**

Later-life health in England has not improved uniformly. Gains in functioning, biomarkers, and cognition coexist with rising pain and poorer mental health. Trends were also socially and age patterned, producing increasingly multidimensional and socially patterned health outcomes. Multidomain health monitoring is essential for interpreting population health trends and planning healthy ageing, prevention, long-term care, and work policies.

**Key messages:**

**What is already known on this topic**

Previous studies have reported mixed trends in later-life health, often focusing on single health domains and giving limited attention to differences by age, education, or measurement method.

**What this study adds**

Between 2004/05 and 2023/24, health at older ages in England changed in divergent rather than uniformly favourable directions, with improvements in mobility, selected biomarkers, and memory alongside worsening pain, diabetes, mental health, and quality of life. Trends also differed by age and education, and self-reported and biomarker-defined cardiometabolic measures sometimes moved in opposite directions.

**How this study might affect research, practice or policy**

Monitoring and planning for ageing populations should use multidomain health measures and consider social inequalities rather than relying on population averages, chronological age, or single indicators alone.

## Full Text Availability

The license terms selected by the author(s) for this preprint version do not permit archiving in PMC. The full text is available from the preprint server.

